# Effect of phosphodiesterase type 5 inhibitors on surgical outcome of ventricular septal defect and pulmonary hypertension patients

**DOI:** 10.1186/s43044-024-00475-5

**Published:** 2024-05-21

**Authors:** Khaled Ahmed Shams, Dalia Monir Ellahony, Ahmed Fouad Halima, Rania Salah Elzayat

**Affiliations:** 1 Cardiology Department, Faculty of Medicine, Helwan University, Helwan, Egypt; 2 Adult Cardiology Department, Aswan Heart Centre, Magdi Yacoub Foundation, Aswân, Egypt; 3 Department of Pediatrics, Faculty of Medicine, Menoufia University, Shebein Elkom, Menoufia Egypt

**Keywords:** Ventricular septal defect, Pulmonary arterial hypertension, Phosphodiesterase type 5 inhibitors, Right ventricle functions, Postoperative pulmonary hypertension, Pulmonary hypertensive crisis

## Abstract

**Background:**

Children with ventricular septal defect (VSD) and large systemic-to-pulmonary shunts eventually develop pulmonary hypertension (PH). The perioperative management of patients with VSD and PH is quite troublesome and still debatable, especially in developing countries where the different management options and standardization of treatment is not available. Oral phosphodiesterase type 5 (PDE-5) inhibitors are good treatment options being widely available, cheap, easy administration and do not require extensive monitoring. The aim of our study was to evaluate the effect of the PDE-5 inhibitors when given orally, early preoperative and continued for 3 months postoperative on controlling postoperative PH with its effect on right ventricle (RV) functions. Fifty-one patients were randomly assigned to either sildenafil or tadalafil, 1 week before and continued for 3 months after corrective surgery. The control group received a placebo.

**Results:**

There was no significant difference in the improvement in the right ventricle systolic pressure (RVSP) between both groups, early in the postoperative period (*P* = 0.255) and in follow-up (*P* = 0.259). There was also no significant difference in the changes in mean pulmonary artery pressure (mPAP), postoperatively and on follow-up (*P* = 0.788 and 0.059, respectively). There was a drop in RV functions in both groups postoperatively which improved on follow-up; however, it was not significant between both groups. The length of intensive care unit (ICU) stay was similar between both groups (*P* = 0.143).

**Conclusion:**

Perioperative administration of PDE-5 inhibitors does not have an impact on the clinical course as regards improvement in pulmonary artery (PA) pressure, ventricular functions and ICU stay.

## Background

Ventricular septal defect (VSD) is one of the most common congenital heart diseases (CHDs) with an incidence of 40% [1]. Significant proportion of patients with VSDs had significant left to right shunting which eventually leads to PH of variable severity. This inevitable increase in pulmonary artery pressure (PAP) had a deleterious effect on both morbidity and mortality, being one of the major determinants of perioperative outcome, as well as long-term well-being and survival [2].

PH due to CHDs is classified as a type of pulmonary arterial hypertension (PAH) [3]. According to the latest European society of cardiology (ESC) guidelines, PAH has been redefined by a mPAP > 20 mmHg at rest together with pulmonary artery wedge pressure (PAWP) < 15 mmHg and pulmonary vascular resistance (PVR) > 2 Wood unit (WU) [3].

The prognosis of VSDs depends to a great extent on the degree of left to right shunting with the resultant histologic changes in the pulmonary vasculature and PVR [4]. Not only is the prognosis badly affected but also the surgical outcome [4]. This urges early surgical correction and development of several treatment algorithms to prevent postoperative sequelae of PH [5].

Pulmonary vascular disease resulting from over-circulation with the resultant pulmonary hypertension may be the most preventable cause worldwide [6]. And so, efforts should be directed toward: solidifying a better understanding of the pathophysiology of pulmonary vascular disease, early detection and management of such condition, offering different types of medical treatment options and ensure availability to personal in less privileged countries, having an evidence-based guidelines for the management of pulmonary hypertension in this peculiar subgroup of patients, taking into consideration the inherent difficulties in conducting clinical research in those patients and having data on long-term outcomes many years after surgical repair [6].

Of the major burdens in the early postoperative period are the sudden rise in pulmonary artery pressure (PAP) and pulmonary hypertensive crisis [7]. Pulmonary hypertensive crisis is a lethal condition characterized by sudden severe rise in PAP leading to profound RV failure, reduction in cardiac output and death [7]. Pulmonary hypertensive crisis is believed to be due to endothelial dysfunction and decrease endogenous nitric oxide (NO) production resulting in decline in the synthesis of cyclic guanosine monophosphate (cGMP) which is a potent pulmonary arterial vasodilator [8].

A profound effort is being implemented to prevent and treat the drastic changes of left to right shunting on the pulmonary vasculature and avoid the unpleasant complications of increased PA pressure on both morbidity and mortality in the early postoperative period. Of those measures are mechanical ventilation, hyperventilation, deep sedation and use of selective pulmonary vasodilators as endothelin receptor antagonists, aerosolized and intravenous prostacyclin, inhaled nitric oxide (iNO) and PDE-5 inhibitors either alone or combined [9].

Despite being quite helpful in managing patients with perioperative PH and pulmonary hypertensive crisis, some of these therapies are not commonly used due to various factors, including unavailability, cost, need for close monitoring, systemic side effects and rebound PH [10].

Nowadays, iNO is the gold standard for the management of postoperative PH and pulmonary hypertensive crisis. Despite being effective, it is expensive, not widely available, especially in developing countries, its administration mandates the presence of special equipment which is not available in all centers and its withdrawal results in rebound PH.

Oral PDE-5 inhibitors are widely available agents that prevent cGMP degradation by inhibiting phosphodiesterase, increasing its plasma levels promoting PA vasodilation. Moreover, they are endothelium independent, administered orally, are well tolerated with very few drug interactions and do not require intensive monitoring, facilitating their utility [11]. Adding on to this, there are different agents available nowadays with long half-life, improving the compliance. It is of importance to mention that administration of PDE-5 inhibitors preoperatively is not without danger, and there might be an extensive increase in the preexisting left to right shunt with its deleterious effect provoking heart failure.

In developing countries, management of patients with VSDs and pulmonary hypertension is extremely challenging, in which nearby medical service is deficient with the resultant delayed presentation, and limited resources making availability of expensive management options scanty. Therefore, we aimed to study the effect of the inexpensive oral PDE-5 inhibitors being sildenafil and tadalafil when given orally, early preoperative and continued for 3 months postoperative on controlling postoperative pulmonary hypertension and crisis with its effect on RV functions.

## Methods

This study was a prospective, randomized, double-blinded, single-center study conducted at a tertiary cardiac referral center, after obtaining the ethical approval from the ethical committee of faculty of Medicine, Menoufia University (IRB approval number: 10/2022 PDEI 11-2), and an informed parental consent was obtained for each patient.

**Fig. 1:**
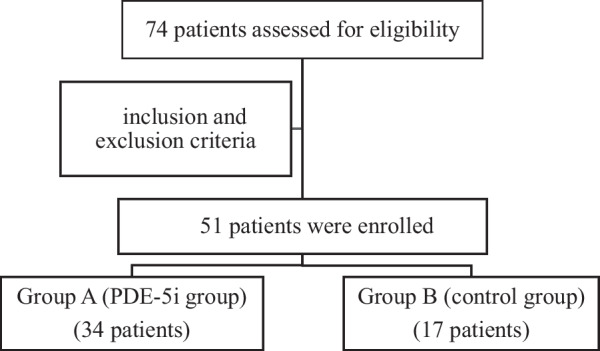
Study population flowchart

Out of 74 patients assessed for eligibility, 51 patients with a definite diagnosis of VSD and pulmonary hypertension were enrolled in the study. The diagnosis of VSD and the severity of pulmonary arterial hypertension were assessed by two-dimensional color Doppler echocardiography. Pulmonary hypertension was defined as having a mPAP > 20 mmHg. Patients with large VSDs and bidirectional shunting, severe pulmonary hypertension and near systemic PAP underwent hemodynamic cardiac catheterization preoperatively to ensure complete reversibility of the PAP with PVR < 4 WU. Patients with the following criteria were excluded from this study: Eisenmenger syndrome, thyroid dysfunction, complete atrioventricular septal defect and cardiac arrhythmias (Fig. 1).

The patients were allocated to treatment or placebo using a computer-generated randomization table. Group A received either sildenafil (0.5 mg/kg/TDS) or tadalafil (1 mg/kg/OD) which were initiated 1 week before and continued for 3 months after corrective surgery. The drugs were administered through nasogastric tube in the early postoperative period till initiation of oral feeding. Group B received placebo.

All patients were managed postoperatively according to the policy of our center with milrinone, dobutamine and diuretics till stabilization of hemodynamics and weaning of ventilation. Patients were kept on anti-failure measures according to their clinical need with monitoring of clinical data inside pediatric ICU (PICU) and after hospital discharge for any drug side effects.

### Echocardiography

All patients underwent three echocardiographic studies: preoperative (1 week before surgery), postoperative (1 week after) and follow-up (3 months after surgery). All echocardiographic studies were performed using an ultrasound system (GE Vivid S5) by the same pediatric cardiologist who was blinded to the study group. Standard 2D and M-mode echocardiograms were obtained according to the American Society of Echocardiography guidelines [12]. Basic measurements included left ventricle (LV) wall thickness, LV internal dimensions, LV end-diastolic and end-systolic volumes, LV EF by M-mode and modified Simpson’s rule, RV end-diastolic and end-systolic areas, right atrial volume and area, RV fractional area change (FAC) (RV FAC > 35% is considered normal) and tricuspid annular plane systolic excursion (TAPSE > 17 mm is considered normal). Doppler measurements included estimated RVSP and mPAP. Pulsed-wave tissue Doppler assessment of the lateral tricuspid annulus to assess RV systolic function (S′ wave velocity > 9.5 cm/s is considered normal) was performed.

The primary outcomes were improvement in RVSP and mPAP. The secondary outcomes were changes in RV functions and ICU hospital stay.

### Statistical analysis

Data were collected and statistically analyzed using SPSS (Statistical Package for Social Science) program version 13 for windows. Qualitative variables were expressed as frequency and percentage. Quantitative variables were expressed as mean ± SD. Qualitative variables were compared by the use of Chi-square test. Quantitative variables were assessed with the unpaired t test. A value of *P* ≤ 0.05 was considered significant, and *P* ≤ 0.001 was considered highly significant.

## Results

Fifty-one patients were enrolled in the study and randomized to either PDE-5 inhibitors (Group A—34 patients) or placebo (Group B—17 patients). There was no fall out seen during the study due to either cardiac death, death from any cause, any other reason or consent withdrawal in the control group (group B). However, there were 2 mortalities in Group A: One patient died from brady/asystole 8 days after surgery and the other patient died 2 weeks after surgery due to subarachnoid hemorrhage; one patient required re-intubation due to pulmonary hypertensive crisis in group A. The rest of both groups continued till the end of the study.

### Demographic characteristics

Both the groups were comparable with no significant difference between the baseline characteristics (Table 1). The age in the PDE-5i group ranged from 3.5 to 24 months and ranged from 4 to 21 months in the control group (*P* = 0.375), and seventeen patients (53.1%) in the PDE-5i and 9 patients (52.9%) in the control group were males (*P* = 0.990). The mean body surface area in the PDE-5i group was 0.33 ± 0.05 m^2^ compared with 0.32 ± 0.04 m^2^ in the control group (*P* = 0.881). There was no significant difference in the pediatric ICU stay between both groups with an average of 3–4 days.

**Table 1:** Demographic data of the study population

Variable		PDEi group (Group A—n = 32)	Control group (Group B—n = 17)	*P* value
Age (months)	Median (IQR)	10 (3.5–24)	9 (4–21)	0.375
Sex	Male	17 (53.1%)	9 (52.9%)	0.990
	Female	15 (46.9%)	8 (47.1%)	
Height (cm)	Mean ± SD	65.66 ± 5.45	67.12 ± 5.07	0.365
Weight (kg)	Mean ± SD	6.34 ± 1.57	6.03 ± 1.11	0.476
Body surface area	Mean ± SD	0.33 ± 0.05	0.32 ± 0.04	0.881
ICU stay	Median (IQR)	4 (2–15)	3 (2–5)	0.143

Data are represented as median (interquartile range), number (%) or mean (SD)

### Baseline echocardiographic characteristics

There was no significant difference as regards LV functions measured by both M-mode and modified Simpson’s rule, RV functions as measured by TAPSE, FAC and tissue Doppler. The baseline RVSP and mean PAP were not statistically different between both groups (Table 2).

**Table 2:** Baseline echocardiographic characteristics

Baseline echocardiographic characteristics		PDE-5i group (Group A–n = 32)	Control group (Group B–n = 17)	*P* value
LVEDDi (mm/m^2^)	Mean ± SD	94.87 ± 19.15	85.59 ± 13.90	0.084
LVESDi (mm/m^2^)	Mean ± SD	58.11 ± 15.12	52.81 ± 9.08	0.193
LVFS (%)	Mean ± SD	40.28 ± 6.84	37.94 ± 5.47	0.230
LV EF M-mode (%)	Mean ± SD	71.19 ± 8.26	70.18 ± 6.05	0.659
LVEDVi (ml/m^2^)	Mean ± SD	89.19 ± 33.69	76.46 ± 29.89	0.252
LVESVi (ml/m^2^)	Mean ± SD	37.37 ± 17.94	27.09 ± 11.10	0.037
LVEF by modified Simpson’s (%)	Mean ± SD	61.56 ± 7.18	63.76 ± 9.32	0.362
SVi	Mean ± SD	61.70 ± 28.57	48.33 ± 22.96	0.103
RVEDAi (mm)	Mean ± SD	25.62 ± 13.61	20.70 ± 5.08	0.159
RVESAi (mm)	Mean ± SD	15.27 ± 8.48	11.79 ± 2.36	0.105
FAC (%)	Mean ± SD	40.06 ± 9.77	41.59 ± 6.42	0.565
TAPSE (mm)	Mean ± SD	17.81 ± 3.04	16.06 ± 2.88	0.057
RVSP (mmHg)	Mean ± SD	70.84 ± 15.93	69.21 ± 17.48	0.769
mPAP (mmHg)	Mean ± SD	46.32 ± 10.93	43.56 ± 12.92	0.445
S′ wave velocity *w* (cm/s)	Mean ± SD	10.88 ± 3.00	10.00 ± 2.15	0.293

LVEDDi, LV end-diastolic dimension indexed; LVESDi, LV end-systolic dimension indexed; FS, Fractional shortening; EF, Ejection fraction; LVEDVi, LV end-diastolic volume indexed; LVESVi, LV end-systolic volume indexed; SVi, Stroke volume indexed; RVEDAi, RV end-diastolic area indexed; RVESAi, RV end-systolic area indexed; FAC, Fractional area change; TAPSE, Tricuspid annular plane systolic excursion; RVSP, RV systolic pressure; mPAP, Mean PA pressure. Independent T Test

### Postoperative data in control group and effect of VSD closure on PAP and RV functions

VSD surgical closure resulted in a significant drop in RVSP and mPAP immediately in the postoperative period and continued to decrease over time as shown in Table 3. There was a postoperative drop in RV function as measured by FAC, TAPSE and tissue Doppler S′ wave velocity which improved on follow-up as shown in Table 3.

**Table 3:** Postoperative and follow-up data in the PDE-5i and control group

		Control group (Group B)			*P* value
		Preoperative	Postoperative	Follow-up	
RVSP (mmHg)	Mean ± SD	69.21 ± 17.48	34.94 ± 11.44	26.42 ± 8.35	< 0.001
MPAP (mmHg)	Mean ± SD	43.56 ± 12.92	21.23 ± 5.49	12.00 ± 1.25	0.003
FAC (%)	Mean ± SD	41.59 ± 6.42	38.41 ± 12.79	47.17 ± 8.11	0.002
TAPSE (mm)	Mean ± SD	16.06 ± 2.88	10.59 ± 5.06	13.40 ± 4.73	0.003
S′ wave velocity (cm/sec.)	Mean ± SD	10.00 ± 2.15	5.12 ± 2.74	8.67 ± 1.50	< 0.001

		PDE-5i group (Group A)			*P* value
		Preoperative	Postoperative	Follow-up	
RVSP (mmHg)	Mean ± SD	70.84 ± 15.93	39.93 ± 15.60	30.95 ± 12.17	< 0.001
MPAP (mmHg)	Mean ± SD	46.32 ± 10.93	22.00 ± 8.94	16.00 ± 6.35	< 0.001
FAC (%)	Mean ± SD	40.06 ± 9.77	38.16 ± 10.30	42.61 ± 10.17	0.119
TAPSE (mm)	Mean ± SD	17.81 ± 3.04	10.56 ± 3.98	14.74 ± 3.53	< 0.001
S′ wave velocity (cm/sec.)	Mean ± SD	10.88 ± 3.00	6.88 ± 3.29	8.88 ± 1.75	< 0.001

RVSP, RV systolic pressure; mPAP, Mean PA pressure; FAC, Fractional area change; TAPSE, Tricuspid annular plane systolic excursion. Independent T test. Repeated measures ANOVA test

### Primary outcome

Administration of PDE-5i did not result in a significant difference in the improvement in RVSP in PDE-5i group (39.93 ± 15.60 mmHg) when compared to control group (34.94 ± 11.44 mmHg) in the postoperative period which also continued during follow-up (30.95 ± 12.17 vs 26.42 ± 8.35 mmHg). As regards mPAP, there was no significant difference in improvement in mPAP between PDE-5i group and control group both in the postoperative period (22.00 ± 8.94 mmHg vs 21.23 ± 5.94 mmHg) and on follow-up (16.00 ± 6.35 mmHg vs 12.00 ± 1.25 mmHg) (Table 4).

**Table 4:** PAP in the PDE-5i and control group:

Postoperative period		PDE-5i group (group A)	Control group (group B)	*P* value
RVSP (mmHg)	Mean ± SD	39.93 ± 15.60	34.94 ± 11.44	0.255
MPAP (mmHg)	Mean ± SD	22.00 ± 8.94	21.23 ± 5.49	0.778

Follow-up		PDE-5i group (group A)	Control group (group B)	*P* value
RVSP (mmHg)	Mean ± SD	30.95 ± 12.17	26.42 ± 8.35	0.259
MPAP (mmHg)	Mean ± SD	16.00 ± 6.35	12.00 ± 1.25	0.059

RVSP, RV systolic pressure; mPAP, Mean PA pressure

### Secondary outcome

In addition to control group, there was a drop in RV functions immediately postoperative in the PDE-5i group which improved on follow-up as shown in Table 3. Comparing the effect of PDE-5 inhibitors on the impairment in RV functions in the postoperative period and on follow-up for both groups, there was no significant difference (Table 5).

**Table 5:** RV functions in the PDE-5i and control group

Postoperative period		PDE-5i group (group A)	Control group (group B)	*P* value
FAC (%)	Mean ± SD	38.16 ± 10.30	38.41 ± 23.79	0.940
TAPSE (mm)	Mean ± SD	10.56 ± 3.98	10.59 ± 5.06	0.984
S′ wave velocity (cm/s)	Mean ± SD	6.88 ± 3.29	5.12 ± 2.74	0.066

Follow-up		PDE-5i group (group A)	Control group (group B)	*P* value
FAC (%)	Mean ± SD	42.61 ± 10.17	47.17 ± 8.11	0.188
TAPSE (mm)	Mean ± SD	14.74 ± 3.53	13.40 ± 4.73	0.351
S′ wave velocity (cm/s)	Mean ± SD	8.88 ± 1.75	8.67 ± 1.50	0.727

FAC, Fractional area change; TAPSE, Tricuspid annular plane systolic excursion. Independent T test

### Subgroup analysis

On subgroup analysis, there was a significant improvement in the RVSP and mPAP in the sildenafil group compared to the tadalafil group; however, there was no significant difference between the two groups regarding the RV functions (Table 6).

**Table 6:** Subgroup analysis between sildenafil and tadalafil

Postoperative period		Sildenafil group	Tadalafil group	*P* value
RVSP (mmHg)	Mean ± SD	34.06 ± 9.46	46.64 ± 18.67	0.025
mPAP (mmHg)	Mean ± SD	17.88 ± 6.27	29.00 ± 8.63	0.001
FAC (%)	Mean ± SD	39.89 ± 10.88	35.93 ± 9.41	0.288
TAPSE (mm)	Mean ± SD	10.39 ± 4.41	10.79 ± 3.51	0.984
S′ wave velocity (cm/s)	Mean ± SD	6.33 ± 2.72	7.57 ± 3.90	0.066

RVSP, RV systolic pressure; mPAP, Mean PA pressure; FAC, Fractional area change; TAPSE, Tricuspid annular plane systolic excursion. Independent T test

## Discussion

Nearly a century and a half after the description of VSDs and perfection of the technique of open surgical repair and the further refinement since then, infants and children who undergo surgical closure of their VSDs are provided excellent outcomes in the proper hands [13, 14]. Consequently, more attention has been directed toward further improving perioperative outcomes, long-term quality of life and survival.

In the early postoperative period, the management of children with VSDs and PH is quite troublesome when persistent PH and pulmonary hypertensive crisis are a major burden on surgical outcome, especially in developing countries where VSD repair may be delayed, and perioperative management is not standardized. Over the past decade, there was a major advance in the treatment of postoperative PH using different agents aiming to ameliorate PH severity and eliminate the effect of persistent PH and improving surgical outcome.

We sought to study the effect of administering oral PDE-5 inhibitors preoperatively and postoperatively on the outcome, anticipating that it might improve both RVSP and mPAP, decreasing pulmonary hypertensive crisis events and total ICU hospital stay with its effect on surgical outcome.

In our study, preoperative initiation of PDE-5 inhibitors did not result in significant decrease in RVSP and mPAP compared to the control group, nor a difference was noted in ICU stay. Of note, there was a significant drop in RV functions immediately postoperative that was not confined to the PDE-5 inhibitors group and occurred in control group as well.

Not surprisingly, many studies have shown that early closure of VSDs results in decline in the peak pulmonary to systemic pressure ratio compared to the preoperative indices immediately after closure, and these results persist on follow-up [15, 16].

Several studies have been conducted to investigate the value of prophylactic use of PDE-5 inhibitors and identify proper timing for administration, using different application schedules either preoperative or immediately postoperative with different and controversial outcomes [8, 11, 17, 18]

In concordant with our study, Hofer et al. [8] showed in a prospective randomized study that postoperative prophylactic application of sildenafil in VSD patients could not influence the clinical course specifically the sudden increase in pulmonary arterial pressure. Other studies [11, 17] could not confirm the superiority of sildenafil even when administered preoperatively, either 2 weeks [11] or 24–8 h [17] before surgery.

On the contrary side, Palma et al. [18] showed that application of sildenafil 1 week before and 1 week after surgery significantly lowered pulmonary arterial pressure in contrast to postoperative administration only with the resultant shortened mechanical ventilation time, and the lengths of ICU and hospital stay. Similar results were demonstrated by Bigdelian and his colleague, and they concluded that preoperative administration of sildenafil is safe to prevent postoperative PH and pulmonary hypertensive crisis and has impact on postoperative care [19].

Our results might have been affected by the fact that absorption could be diminished in the early postoperative period due to decreased intestinal motility, decreasing the drug bioavailability, unfortunately the IV form is not available in our country, and we were mainly aiming of studying an agent that is easy to administer. Kesvani et al. [20] showed in their prospective randomized study that intravenous administration of sildenafil resulted in significant reduction in pulmonary artery systolic pressure. This was also confirmed in another study done by Sharma and his colleagues [21].

On subgroup analysis, sildenafil was found to be more effective in decreasing RVSP and mPAP compared to tadalafil; however, the number of patients in both groups was too small to extrapolate data from and optimal dosing of tadalafil in young infants is still questionable. Sabri et al. in their randomized controlled trial comparing sildenafil to tadalafil when administered preoperative and postoperative in 42 patients with large VSDs and PH, and they concluded that tadalafil has an appropriate efficacy and safety profile as sildenafil in young infants [22].

One important finding of this study was that both right and left ventricular functions were significantly reduced postoperatively. Our finding is consistent with the results reported by Vassalos and his colleagues [17] where they reported a drop in ventricular functions after corrective surgery. They suggested administration of sildenafil to be the cause which is not consistent with our results and further mechanism for such condition should be explored.

The sensitivity of the RV to afterload changes is more than the left ventricle. Several factors have been proposed including prolonged cardiopulmonary bypass time, residual PAH and rebound PH [17]. However, those mechanisms are not supported by the study findings of drop of both right and left ventricular functions where the LV will not be affected by PA pressure changes. Another possible mechanism is administration of PDE-5 inhibitors itself [17], through increasing cGMP that can suppress contractility by decreasing myocardial calcium sensitivity, blunting the contractile response to adrenergic effect [23, 24]. However, this mechanism is not supported by our study finding of drop of ventricular functions in control group as well as PDE-5 inhibitors group.

The limitations of this study are small population size, short follow-up duration and absence of IV form of sildenafil.

## Conclusion

Preoperative and postoperative administration of PDE-5i does not have an impact on the clinical course of the patients, especially in the early postoperative period as regards improvement in PA pressure, ICU stay and ventricular functions.

## Data Availability

The datasets used and/or analyzed during the current study are available from the corresponding author on reasonable request.

## Abbreviations

cGMP: Cyclic guanosine monophosphate
CHDs: Congenital heart diseases
FAC: Fractional area change
iNO: Inhaled nitric oxide.
LV: Left ventricle
mPAP: Mean pulmonary artery pressure.
No: Nitric oxide
PA: Pulmonary artery
PAH: Pulmonary arterial hypertension
PAP: Pulmonary artery pressure
PAWP: Pulmonary artery wedge pressure
PDE-5: Phosphodiesterase type 5
PH: Pulmonary hypertension
PVR: Pulmonary vascular resistance
RV: Right ventricle
RVSP: Right ventricle systolic pressure
TAPSE: Tricuspid annular plane systolic excursion
VSD: Ventricular septal defect
WU: Wood unit
